# Human Lysyl Oxidase Over-Expression Enhances Baseline Cardiac Oxidative Stress but Does Not Aggravate ROS Generation or Infarct Size Following Myocardial Ischemia-Reperfusion

**DOI:** 10.3390/antiox11010075

**Published:** 2021-12-29

**Authors:** Laura Valls-Lacalle, Lídia Puertas-Umbert, Saray Varona, José Martínez-González, Cristina Rodríguez, Antonio Rodríguez-Sinovas

**Affiliations:** 1 Cardiovascular Diseases Research Group, Vall d’Hebron Institut de Recerca (VHIR), Vall d’Hebron Barcelona Hospital Campus, 08035 Barcelona, Spain; lvalls92@gmail.com; 2 Centro de Investigación Biomédica en Red de Enfermedades Cardiovasculares (CIBER-CV), Instituto de Salud Carlos III, 28029 Madrid, Spain; saray.var@gmail.com (S.V.); jose.martinez@iibb.csic.es (J.M.-G.); 3 Institut de Recerca del Hospital de la Santa Creu i Sant Pau, 08041 Barcelona, Spain; lpuertas@santpau.cat; 4 Instituto de Investigación Biomédica Sant Pau, 08041 Barcelona, Spain; 5 Instituto de Investigaciones Biomédicas de Barcelona-Consejo Superior de Investigaciones Científicas (IIBB-CSIC), 08036 Barcelona, Spain

**Keywords:** lysyl oxidase, reperfusion injury, cardiac remodeling

## Abstract

Lysyl oxidase (LOX) is an enzyme critically involved in collagen maturation, whose activity releases H_2_O_2_ as a by-product. Previous studies demonstrated that LOX over-expression enhances reactive oxygen species (ROS) production and exacerbates cardiac remodeling induced by pressure overload. However, whether LOX influences acute myocardial infarction and post-infarct left ventricular remodeling and the contribution of LOX to myocardial oxidative stress following ischemia-reperfusion have not been analyzed. Isolated hearts from transgenic mice over-expressing human LOX in the heart (TgLOX) and wild-type (WT) littermates were subjected to global ischemia and reperfusion. Although under basal conditions LOX transgenesis is associated with higher cardiac superoxide levels than WT mice, no differences in ROS production were detected in ischemic hearts and a comparable acute ischemia-reperfusion injury was observed (infarct size: 56.24 ± 9.44 vs. 48.63 ± 2.99% of cardiac weight in WT and TgLOX, respectively). Further, similar changes in cardiac dimensions and function were observed in TgLOX and WT mice 28 days after myocardial infarction induced by transient left anterior descending (LAD) coronary artery occlusion, and no differences in scar area were detected (20.29 ± 3.10 vs. 21.83 ± 2.83% of left ventricle). Our data evidence that, although LOX transgenesis induces baseline myocardial oxidative stress, neither ROS production, infarct size, nor post-infarction cardiac remodeling were exacerbated following myocardial ischemia-reperfusion.

## 1. Introduction

Acute myocardial infarction as a result of coronary atherothrombotic disease is the main common cause of death and disability worldwide. Early restoration of blood flow by percutaneous coronary intervention (PCI) is the gold-standard therapeutic strategy in patients with STEMI (ST-segment elevation myocardial infarction), limiting infarct size and, thereby, preserving cardiac function and improving clinical outcomes. Unfortunately, revascularization promotes the so-called myocardial reperfusion injury, which is associated with an exaggerated reactive oxygen species (ROS) production that contributes to cardiomyocyte damage and death [1,2].

Cardiac repair after myocardial infarction depends on a highly coordinated response that entails the sequential recruitment and removal of inflammatory and mesenchymal cells [3]. Initially, alarmins secreted by necrotic cardiomyocytes induce an intense inflammatory reaction, resulting in the removal of dead cells and matrix debris. This is followed by a proliferative phase, associated with suppression of inflammation, and characterized by tissue infiltration of mesenchymal cells responsible for the extracellular matrix (ECM) protein secretion. During maturation, reparative cells are removed and matrix cross-linking occurs. Due to the limited regenerative capacity of the adult mammalian heart, this reparative fibrotic response is the only way to preserve cardiac structure and integrity, avoiding rupture and maintaining pumping capacity.

The ECM does not merely provide a structural scaffold for cells and tissues, but it influences essential cellular functions and signaling [4,5]. In fact, the disturbance of ECM composition/structure following myocardial infarction boosts inflammation and repair [6] and substantially contributes to cardiac dysfunction. The principal structural component of the myocardial ECM is collagen, which is mainly synthesized by fibroblasts and myofibroblasts [7]. Collagen deposition progressively leads to adverse cardiac remodeling, increased tissue stiffness and diastolic dysfunction, reduced contractility, and eventually heart failure (HF). However, an increase in collagen deposition does not always directly translate into enhanced stiffness, as myocardial injury results in increased collagen content, but also in qualitative changes in its structure, including post-translational modifications [8,9]. In this regard, lysyl oxidase (LOX)-dependent collagen cross-linking has been shown to be a major factor influencing cardiac stiffness during remodeling [10,11,12]. Covalent collagen and elastin cross-links are catalyzed by LOX, a copper-dependent extracellular amino oxidase [13,14]. LOX oxidizes peptidyl lysines to highly reactive semi-aldehydes, which condense with neighboring amino or peptidyl groups resulting in intra- and inter-molecular covalent cross-links and producing H_2_O_2_ as a by-product [13,14].

Previous studies have suggested that LOX may have a detrimental effect on cardiac remodeling. Thus, in patients with hypertensive HF, a high degree of cardiac collagen cross-linking is associated with cardiac stiffness and elevated filling pressures [10]. Further, a negative correlation between the activity of LOX and cardiac function was observed in adult male rats submitted to aortocaval fistula-induced volume overload [15], in which the pharmacological inhibition of LOX prevented cardiac dysfunction and collagen accumulation [15]. Similarly, we have recently demonstrated that LOX over-expression promotes an age-dependent concentric remodeling of the left ventricle, impairs diastolic function and aggravates angiotensin II (AngII)-induced cardiac hypertrophy and dysfunction, resulting in an extensive fibrotic response, with a pronounced collagen deposition and cross-linking [16]. Likewise, cardiac LOX over-expression is associated with an excessive generation of both basal and AngII-induced production of ROS. Despite these findings, the impact of LOX on remodeling after myocardial infarction and the contribution of LOX-derived ROS to this process has not been previously investigated. As infarct size is a major determinant of cardiac remodeling and scar formation, here, we first assessed the effect of LOX on acute myocardial ischemia-reperfusion injury in isolated hearts from a transgenic mouse model that over-expresses LOX in the myocardium. Subsequently, the impact of human LOX transgenesis on myocardial function and scar size was analyzed by echocardiography and picrosirius red staining, respectively, 28 days after transient coronary occlusion.

## 2. Materials and Methods

### 2.1. Animals

Studies were performed in TgLOX mice, in which the human LOX cDNA transgene is driven by the CAG promoter (human cytomegalovirus [CMV] immediate early promoter enhancer with chicken β-actin/rabbit β-globin hybrid promoter) [16]. Non-transgenic littermates (wild-type; WT) on a C57BL/6J genetic background were used as controls. Mice were bred in the Animal Experimentation Unit (Institut de Recerca de l’Hospital de la Santa Creu I Sant Pau [IRHSCSP], Barcelona, Spain). The study was performed according to the Spanish Policy for Animal Protection RD53/2013, which meets the European Union Directive, 2010/63/UE and the NIH Guide for the Care and Use of Laboratory Animals (NIH publications N°. 85–23, revised 1996, updated in 2011). Experimental procedures were approved by both the IRHSCSP and the Vall d’Hebron Research Institute ethical committees (reference 35/17 CEEA).

### 2.2. Ischemia-Reperfusion Injury in Isolated Mouse Hearts

TgLOX and WT (C57BL/6J) adult male mice (12–15 weeks, 25–30 g) were anesthetized by intraperitoneal injection of sodium pentobarbital (1.5 g/kg). Hearts were exposed by a bilateral thoracotomy and quickly excised. A retrograde aortic perfusion of the heart was carried out with an oxygenated (95% O_2_: 5% CO_2_) Krebs solution at 37 °C (in mmol/L: NaCl 118, KCl 4.7, MgSO_4_ 1.2, CaCl_2_ 1.8, NaHCO_3_ 25, KH_2_PO_4_ 1.2, and glucose 11, pH 7.4) in a constant flow Langendorff system [17]. First, the flow was adjusted to yield a perfusion pressure of 80–90 mmHg (normoxic conditions). Left ventricular (LV) pressure was monitored using a water-filled latex balloon coupled to a pressure transducer inserted into the left ventricle. Then, the volume of the balloon was adjusted to obtain a LV end-diastolic pressure (LVEDP) between 6 and 8 mmHg. All signals were registered using a ML119 PowerLab interface and Chart 5.0 software (AdInstruments, Castle Hill, Australia).

#### 2.2.1. Experimental Protocol

Hearts from TgLOX and WT mice were equilibrated for 30 min and then submitted to 35 min of global ischemia, followed by a 60 min reperfusion period. During the ischemic phase, hearts were immersed in hypoxic Krebs solution at 37 °C in which glucose was replaced by sucrose (bubbled with 95% N_2_: 5% CO_2_). For ROS detection, reperfusion was stopped after 5 min in a specific subgroup of hearts. Infarct size, lactate dehydrogenase (LDH) release, and functional recovery (LVdevP) were determined.

#### 2.2.2. LDH Release and Infarct Size Measurement

The release of LDH was analyzed in the coronary effluent by spectrophotometry at 340 nm, as previously described [18]. At the end of reperfusion cardiac slices were stained with 2,3,5-triphenyltetrazolium to establish the infarct size. Cardiac slices were weighted and photographed using a digital camera. The size of the necrotic area in each slice and total slice area were calculated using Image-Pro Plus (Media Cybernetics Inc., Rockville, MD, USA) software. Final infarct size was expressed as percentage of total cardiac weight taking into consideration the individual weights of each slice [18].

### 2.3. In Situ Mouse Model of Post-Infarct Cardiac Remodeling

TgLOX and WT (C57BL/6J) adult male mice (12–15 weeks, 25–30 g) were anesthetized using ketamine and sodium pentobarbital (50 mg/kg and 40 mg/kg, respectively; IP) [19] and mechanically ventilated (SAR-830/P Ventilator, CWE Inc, Ardmore, PA, USA). Animals were laid on a heating pad adjusted to keep the body temperature between 36 and 37 °C. After a left lateral thoracotomy at the fourth intercostal space, the left anterior descending (LAD) coronary artery was occluded for 45 min using a 6/0 silk suture placed 1 mm distal to the left atrial appendage. Once the period of coronary occlusion was completed, ligature was released, allowing a period of reperfusion of 28 days, as previously described [20]. A small fragment of the silk was kept in its position to allow later location of the point of occlusion. Coronary occlusion was verified visually by pallor color of the area at risk and by ST segment elevation in surface electrocardiogram. The chest was closed in layers and animals were allowed to recover. After surgery, buprenorphine (0.05 mg/kg per 6 h, SC) was administered for the first 48 h.

#### 2.3.1. Transthoracic Echocardiography

The echocardiographic assessment was performed at baseline, 14 days after reperfusion and at the end of the experimental protocol. A Vivid q portable ultrasound system equipped with an ILS 12 MHz transducer (GE Healthcare, Chicago, IL USA) was used and applied to the shaved chest wall of mice anesthetized with isofluorane (1–1.5%). The following parameters were measured by M-mode echocardiography: ejection fraction (EF), left ventricular end-diastolic internal diameter (LVEDD), left ventricular end-systolic internal diameter (LVESD), interventricular septum thickness (IVS) and posterior wall thickness (LVPW). Moreover, fractional shortening (FS) was calculated as (LVEDD-LVESD)/LVEDD × 100.

#### 2.3.2. Measurement of Scar Area and Cardiomyocyte Cross-Sectional Area

Once the experimental procedure was accomplished, animals were sacrificed by a sodium pentobarbital overdose (1.5 g/kg, IP). Hearts were quickly excised, and after removal of both atria and great vessels, ventricles were divided into two parts. The area located basally to the ligature was weighted and frozen in liquid N_2_, whereas the area located apical to the ligature (including the area at risk) was cross-sectioned into three thin slices, which were weighted, fixed with 4% paraformaldehyde (overnight) and embedded in paraffin. Paraffinized slices were cut in 4 μm sections that were stained with picrosirius red (Sigma-Aldrich, St. Louis, MO, USA), automatically scanned (3D Histech Pannoramic Midi slide scanner) and quantified using ImageProPlus software (Media Cybernetics, Inc., Rockville, MD, USA). Scar area was expressed as the percentage of the fibrotic area in the scanned images respect to the left ventricular area, considering the weight of each slice, as previously described. Replacement fibrosis was additionally analyzed at ×200 magnification to determine the area occupied by collagen within the myocardial scar. In addition, interstitial collagen in distant myocardium from the postero-septal region was also determined in six random histological fields at ×200 magnification [21]. Cardiomyocyte cross-sectional area was assessed after staining with FITC-conjugated wheat germ agglutinin (WGA) as described [22]. Quantitative analysis was performed at ×400 magnification, considering eight random fields in each heart and analyzing 10 cells per field. Similar results were obtained when cardiomyocyte cross-sectional area was quantified on hematoxylin- and eosin-stained cardiac sections [21].

### 2.4. Real-Time PCR

Total RNA was isolated from cardiac samples using TriPure Isolation Reagent (Roche Diagnostics, Mannheim, Germany). The High Capacity cDNA Reverse Transcription Kit (Applied Biosystems, Foster City, CA, USA) and random hexamers were used for RNA (500 ng) reverse transcription. mRNA levels were quantified by real-time PCR using primers and probes (provided by Applied Biosystems or Integrated DNA Technologies, Coralville, IA, USA) for human LOX (Hs00942480_m1) and murine TATA-binding protein (TBP; Mm.PT.39a.22214839), used as endogenous control. The ABI PRISM 7900HT sequence detection system instrument (Applied Biosystems, Foster City, CA, USA) was used and relative mRNA levels were determined using the 2^−ΔΔCt^ method, as described [23]. 

### 2.5. Western Blotting

Hearts were homogenized in ice-cold 50 mM HEPES (pH 7.5) containing 100 mM NaCl, 0.5 mM EGTA, 100 mM glycerol-2-phosphate, 10% glycerol, 0.1% Tween 20, supplemented with 1 mM DTT and a protease inhibitor cocktail (Sigma-Aldrich, St. Louis, MO, USA). Lysates were resolved under reducing conditions by sodium dodecyl sulphate-polyacrilamide gel electrophoresis (SDS-PAGE) and then transferred to polyvinylidene difluoride membranes (Immobilon, Merck-Millipore; Burlington MA, USA, IPVH00010). Membranes were incubated with antibodies against LOX (NB-100-2527, Novus Biologicals, Minneapolis, MN, USA), and glyceraldehyde-3-phosphate dehydrogenase (GAPDH; MAB374, Merck Millipore). Detection was carried out using suitable horseradish peroxidase-conjugated secondary antibodies (Dako Products, Agilent, Santa Clara, CA, USA) and the Luminata^TM^ Western HRP Substrate (Immobilon, Merck-Millipore). Protein size was assessed using protein molecular weight standards (Hyperpage Prestained Protein Marker; Bioline, Paris, France). Results were normalized by GAPDH protein levels.

### 2.6. Immunohistochemistry

Heart samples from WT and TgLOX mice were fixed in 4% paraformaldehyde/0.1 M PBS (pH 7.4) for 24 h and embedded in paraffin. Tissue sections were deparaffinized, rehydrated and subjected to antigen retrieval in 10 mM citrate buffer pH 6.0. Then slides were soaked in a 3% hydrogen peroxide solution in methanol for 30 min and blocked with 10% normal serum. Immunostaining was carried out with an antibody against LOX (ab31238, Abcam, Cambridge, UK). After extensive washing, a biotinylated goat anti-rabbit secondary antibody (Vector Laboratories, Burlingame, CA, USA) was added and immunocomplexes were detected using the Vectastain (ABC) avidin–biotin peroxidase complex (Vector Laboratories) and 3,3′-diaminobenzidine (DAB). Finally, sections were counterstained with hematoxylin and mounted. Serial sections in which the primary antibody was excluded were used as negative controls.

### 2.7. ROS Detection

In situ detection of superoxide anion (O_2_^−^) levels was performed in mouse heart sections by dihydroethidium (DHE) staining (Sigma–Aldrich), as previously described [24]. Heart samples were embedded in Tissue Tek OCT embedding medium (Sakura Finetek Europe B.V., Alphen aan den Rijn, The Netherlands), and frozen in liquid nitrogen. Cardiac slices were equilibrated for 30 min at 37 °C in Krebs-HEPES buffer (in mmol/L: NaCl 130, KCl 5.6, CaCl_2_ 2, MgCl_2_ 0.24, HEPES 8.3, and glucose 11, pH = 7.4), incubated with DHE (2 μM), cover-slipped and maintained for 30 min in a humidified chamber at 37 °C in the dark. The fluorescent signal was analyzed with a fluorescent laser scanning confocal microscope (Leica TCS SP5 confocal microscopy (20× for quantification and illustration) and the Leica LAS AF Lite software (Leica Microsystems S.L.U). Fluorescent images were acquired using a 561 nm laser (long-wavelength excitation). Data were expressed as % of signal in control samples to minimize laser fluctuations from one day to another.

### 2.8. Statistics

Data are expressed as mean ± SEM. Differences were considered significant when *p* < 0.05. Statistical analysis were carried out by Student’s *t*-test (infarct size, cumulative LDH release) or repeated measures ANOVA (MANOVA) (changes in LVdevP or perfusion pressure during reperfusion in experiments in isolated mice heart) and Dunnett’s post hoc test. The D’Agostino-Pearson omnibus normality test was applied and subsequently, the Mann–Whitney U test with the Tukey’s post hoc test or the Kruskal–Wallis test with Dunn’s multiple comparison post-hoc test were employed when the normal distribution of data failed. 

## 3. Results

### 3.1. LOX Transgenesis Is Associated with Enhanced Cardiac LOX Protein Levels and ROS Production

**Figure 1:**
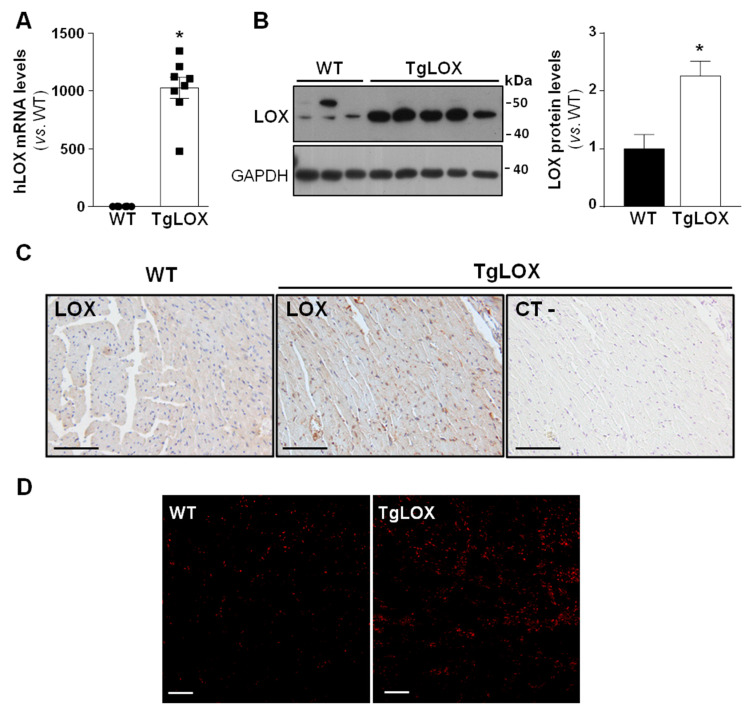
TgLOX mice show increased LOX expression in the myocardium and enhanced ROS production. (**A**) Human LOX (hLOX) mRNA levels were analyzed in hearts from wild-type (WT) and TgLOX mice by real-time PCR. Levels of human LOX mRNA were undetectable in WT mice, but an arbitrary value of 1 was attributed for comparative purposes only (WT, *n* = 6; TgLOX, *n* = 8). (**B**) Protein levels of LOX were assessed by Western blot in heart lysates from WT and TgLOX mice. GAPDH levels are shown as a loading control. Representative immunoblot images have been included. The histogram shows the result from the densitometric analysis (WT, *n* = 4; TgLOX, *n* = 6). Data are expressed as mean ± SEM. * *p* < 0.05 vs. WT. *t*-test (**A**) and Mann–Whitney test (**B**) were applied. (**C**) Immunohistochemical analysis of LOX in hematoxylin counterstained myocardial sections from these animals. A negative control, in which the primary antibody was excluded, was used to test for non-specific binding of the secondary antibody (bars: 100 µm). (**D**) Representative dihydroethidium (DHE) staining in WT and TgLOX mice hearts (bars: 100 µm).

To analyze the impact of LOX on cardiac ischemia-reperfusion injury and remodeling, studies were carried out in a transgenic mouse line that over-expresses human LOX under the control of the CAG promoter, which drives the expression of the transgene preferably into the heart [16,22]. The transgenic line, which was periodically refreshed by backcrossing to the C57BL/6J inbred strain, evidences a consistent expression of the human LOX transgene (Figure 1A). TgLOX mice display a significant increase in cardiac LOX protein levels compared with control littermates, as revealed by Western blot (Figure 1B and Figure S1) and immunohistochemical analyses (Figure 1C). Further, and as shown in Figure 1D, confocal microscopy revealed a stronger fluorescence intensity of DHE in the myocardium from TgLOX mice, supporting that the over-expression of LOX triggers an enhanced generation of superoxide anion.

### 3.2. Impact of Acute Ischemia-Reperfusion Injury on Left Ventricular Function, Infarct Size, and ROS Production in Isolated Mouse Hearts

**Figure 2:**
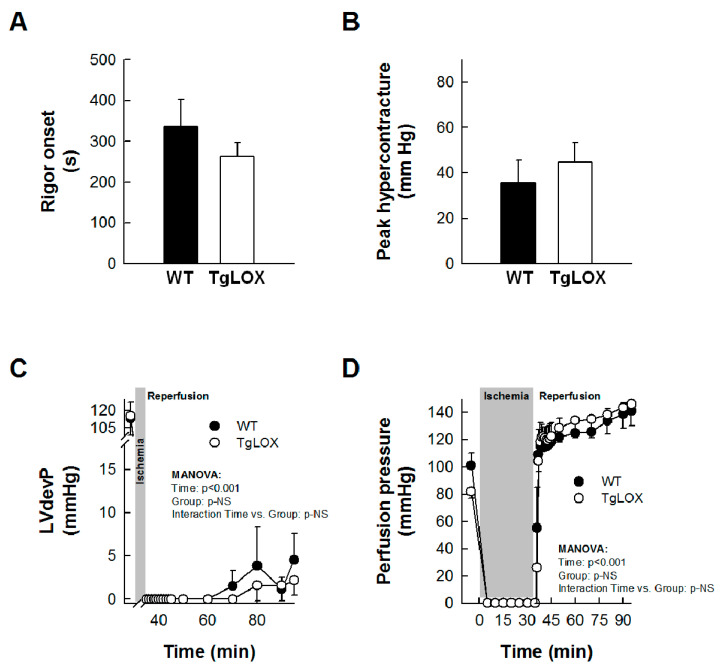
Impact of LOX transgenesis on rigor onset, peak hypercontracture, and functional recovery in isolated mice hearts. Rigor onset during ischemia (**A**) and peak hypercontracture (**B**) during initial reperfusion in isolated wild-type (WT) and TgLOX mice hearts submitted to global ischemia-reperfusion (*n* = 5/group). No significant differences between both groups were observed (Student’s *t*-test). (**C**,**D**) show functional recovery during reperfusion (LVdevP, (*n* = 4/group)) (**C**) and changes in perfusion pressure (**D**) (*n* = 5/group) in isolated hearts from both groups. No significant differences between both groups were observed (repeated measures ANOVA and Dunnett’s tests). Data are expressed as mean ± SEM.

Left ventricular developed pressure (LVdevP) was quickly depressed during ischemia, and was minimal after 2–3 min. An abrupt increase in LVEDP, indicative of ischemic rigor contracture, occurred during the first minutes of ischemia, with no differences between groups (Figure 2A). Reperfusion triggered a new increase in LVEDP, corresponding to hypercontracture, which was not significantly modified in hearts from TgLOX mice (Figure 2B). Similarly, no significant differences were observed between control and TgLOX hearts in functional recovery or perfusion pressure during the entire reperfusion (Figure 2C,D).

**Figure 3:**
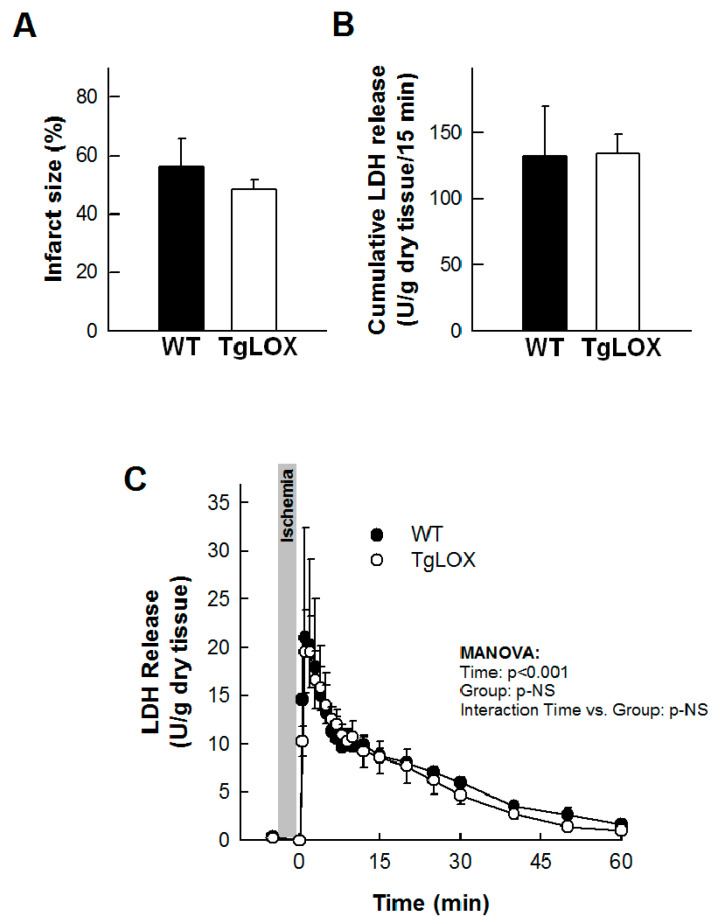
Ischemia-reperfusion injury in isolated mice hearts from wild-type (WT) and TgLOX animals (*n* = 5/group). No differences were observed between both groups in infarct size ((**A**); Student’s *t*-test), cumulative lactate dehydrogenase (LDH) release during the first 15 min of reperfusion ((**B**); Student’s *t*-test) or LDH release during the whole reperfusion period ((**C**); MANOVA). Data are expressed as mean ± SEM.

**Figure 4:**
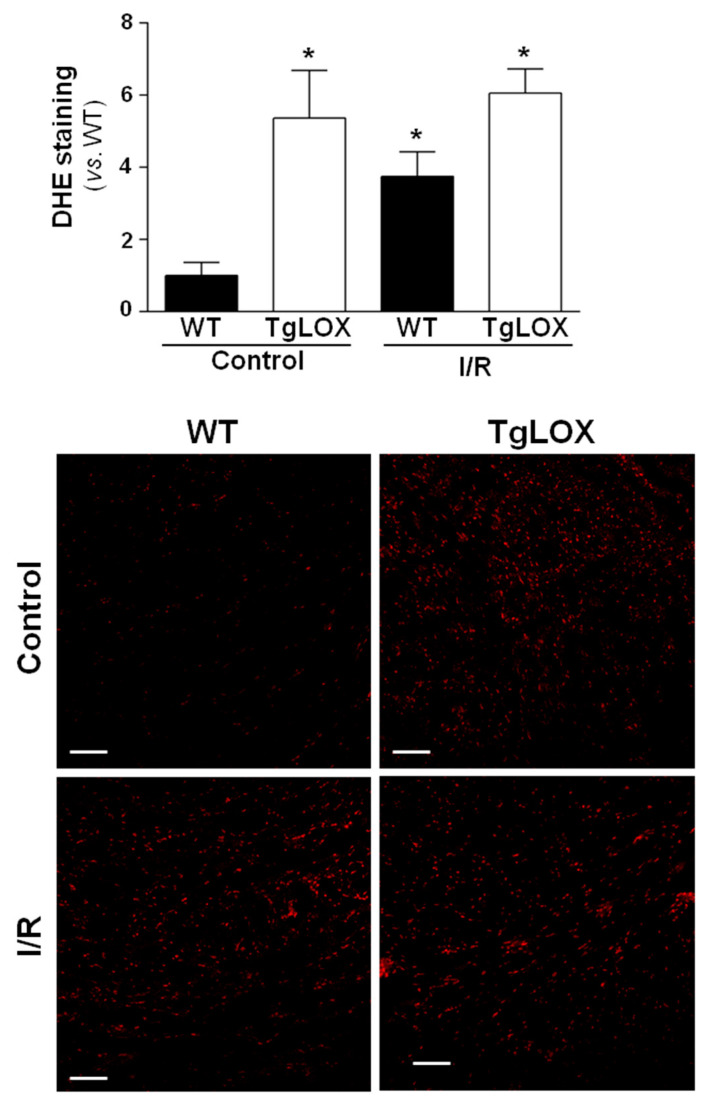
Influence of LOX transgenesis on cardiac reactive oxygen species (ROS) generation after ischemia-reperfusion injury. Superoxide anion levels were assessed by dihydroethidium (DHE) staining in isolated hearts from wild-type (WT) and TgLOX mice subjected to normoxic perfusion (control) or to global ischemia-reperfusion (I/R). DHE staining was quantified at 5 min of reperfusion. Representative images are shown (bars: 100 µm). At least 4 fields from *n* = 3 mice in each group were quantified. Data, are expressed as mean ± SEM. * *p* < 0.05 vs. WT normoxic hearts (Kruskal–Wallis test).

The infarct size averaged 56.24 ± 9.44% in control-isolated hearts subjected to 35 min global ischemia followed by reperfusion, and was not modified in hearts from LOX-over-expressing mice (Figure 3A). Likewise, the release of LDH during reperfusion was also not significantly different between both groups of animals (Figure 3B,C). Further, myocardial ROS levels were analyzed by DHE staining. Consistent with previous reports, acute ischemia-reperfusion injury led to an increase in superoxide production in WT hearts. In contrast, ischemia-reperfusion did not further enhance DHE staining in samples from TgLOX mouse hearts, which was already elevated under basal conditions (Figure 4).

### 3.3. Effect of Human LOX Over-Expression on Mice Survival and Left Ventricular Remodeling in Response to Myocardial Infarction

**Figure 5:**
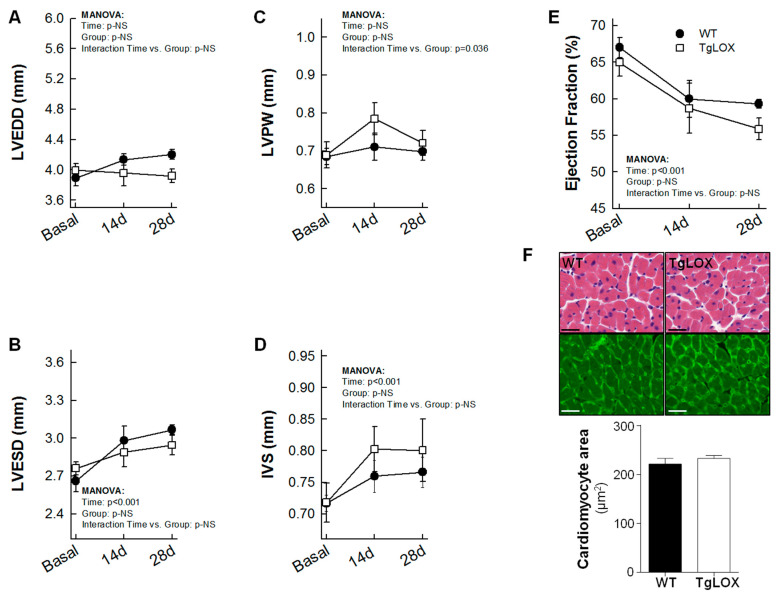
Changes in left ventricular end-diastolic internal diameter (LVEDD; (**A**)), left ventricular end-systolic internal diameter (LVESD; (**B**)), posterior wall thickness (LVPW; (**C**)), interventricular septum thickness (IVS; (**D**)), ejection fraction (**E**) and cardiomyocyte cross-sectional area assessed on WGA stained sections (**F**) in wild-type (WT; *n* = 6) and TgLOX (*n* = 7) mice subjected to a brief period of coronary artery occlusion (45 min) and 28 days of reflow. Representative images of hematoxylin-eosin (upper panels) and WGA (lower panels)-stained sections are shown in (**F**) (bars: 25 µm).

Thirteen out of 18 mice subjected to the protocol of transient coronary occlusion survived (27.78% mortality), although survival rate was not different between control (6 out of 9) and TgLOX (7 out of 9) mice. Myocardial infarction resulted in a progressive and significant increase in left ventricular end-systolic volume and in IVS thickness both in control and TgLOX mice, 28 days after reperfusion, with no differences between groups (Figure 5A–D). Moreover, ejection fraction was similarly depressed in both groups of animals (Figure 5E). In agreement with echocardiographic data, the cardiomyocyte cross-sectional area, quantified either in sections stained with WGA or in hematoxylin-eosin stained samples, was similar in both groups. (Figure 5F).

**Figure 6:**
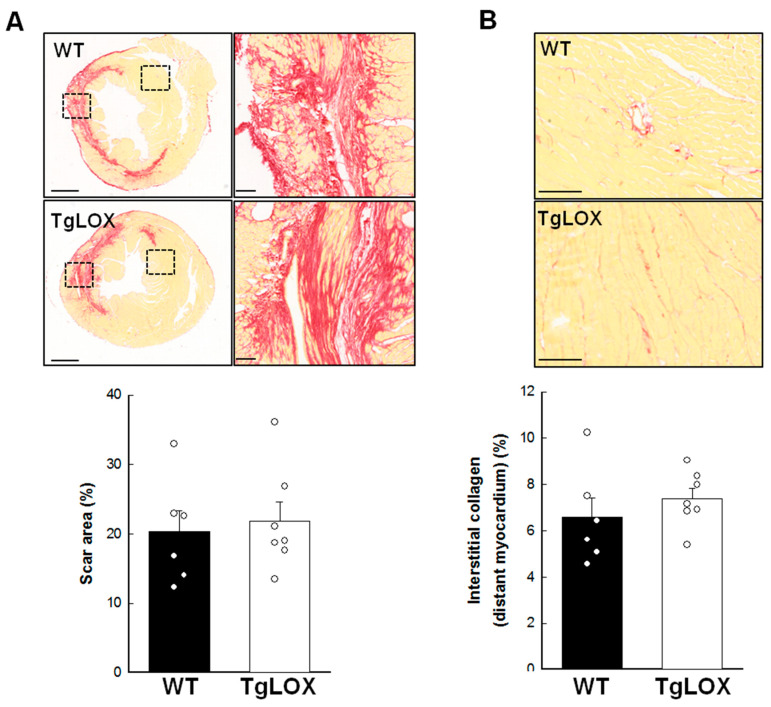
(**A**) Scar area, expressed as percentage of left ventricular (LV) area in the scanned images, in wild-type (WT; *n* = 6) and TgLOX (*n* = 7) mice subjected to a 45-min period of coronary artery occlusion and 28 days of reperfusion. Representative images of myocardial sections stained with picrosirius red are shown for each group (bars: 1000 µm). Squared areas magnified on the right evidence collagen content in scar area (bars: 100 µm). (**B**) Interstitial collagen deposition in remote myocardium. Representative images are shown (bars: 100 µm). No significant differences between both groups were observed (Student’s *t*-test).

Scar area, measured as the area of fibrosis in the scanned images from the infarcted hearts 28 days after reperfusion, was 20.29 ± 3.10% of the left ventricular area, and was not modified by LOX transgenesis (Figure 6A). Collagen represented 68.47 ± 5.49 and 59.06 ± 3.17% of scar area in WT and TgLOX animals, respectively, with no differences between groups (Figure 6A, magnified panels). Remaining space was occupied by surviving cardiomyocytes and vessels, among others. In addition, interstitial collagen deposition in remote myocardium, analyzed by picrosirius red-staining and conventional microscopy, was comparable in both groups of animals (Figure 6B; 6.57 ± 0.85% in control animals vs. 7.38 ± 0.45% in TgLOX mice, p-NS).

## 4. Discussion

The main aim of this work was to assess the consequence of LOX over-expression on acute ischemia-reperfusion injury and its impact on cardiac remodeling 28 days post-myocardial infarction. Here, we show that cardiac LOX over-expression increased ROS generation, but it did not significantly alter acute ischemia-reperfusion injury, cardiac function or left ventricular remodeling after myocardial infarction. 

The hallmark of myocardial reperfusion damage is the impairment of redox balance that leads to oxidative stress [1]. Our previous research evidenced that LOX over-expression in cardiac fibroblasts was associated with enhanced levels of H_2_O_2_ [16], a by-product of LOX catalytic activity [13,14]. Further, myocardial LOX over-expression induces an excessive production of both basal and AngII–mediated superoxide production. Therefore, we hypothesized that the enhanced oxidative stress triggered by LOX might increase acute ischemia-reperfusion injury and subsequently aggravate scar formation. Thus, we submitted hearts isolated from both WT and LOX-over-expressing mice to 35 min of global ischemia followed by reperfusion. However, in using this approach, we demonstrated that human LOX over-expression does not modify acute myocardial ischemia-reperfusion injury, as no changes were observed in either infarct size, determined by triphenyltetrazolium staining, LDH release, or functional recovery. We have previously observed a link between up-regulated LOX and enhanced ROS production in cardiac hypertrophy, and cardiovascular remodeling in both obesity and hypertension [16,25,26]. However, although we have consistently shown that cardiac LOX over-expression increases oxidative stress, the sudden reperfusion of ischemic myocardium did not modify cardiac superoxide levels in transgenic mice. Indeed, myocardial ROS levels achieved under ischemic conditions were comparable between WT and TgLOX mice, and this could explain, at least in a part, why no differences in infarct size and functional recovery were observed.

LOX is an extracellular copper-dependent enzyme, mainly synthesized by fibroblasts and myofibroblasts, that catalyzes the formation of lysine and hydroxylysine-derived cross-links in collagen chains, thus being essential to ensure normal assembly and mechanical properties of the ECM [27]. Previous studies suggested that high LOX levels might have a negative influence on cardiac remodeling. Indeed, over-expression of human LOX was associated with an abnormal, age-dependent, pattern of echocardiographic E/A waves [16], indicative of restrictive filling and diastolic dysfunction [28], and with an age-associated concentric remodeling [16]. These findings strengthen the relationship between LOX and diastolic dysfunction that was initially suggested in patients with heart failure [29], in which a high degree of myocardial collagen cross-linking is further associated with an enhanced risk of hospitalization [30]. Our current results, from young animals with normal cardiac function, do not exclude that LOX transgenesis may differently affect post-myocardial infarction scar formation in aged animals.

A negative correlation between LOX activity and cardiac function has also been reported in animal models of cardiac overload [8]. Interestingly, inhibition of LOX activity has been shown to attenuate cardiac dysfunction and collagen accumulation induced by volume overload in a rat model of aortocaval fistula [15]. Moreover, LOX over-expression has been shown to result in greater fibrotic responses and increased collagen cross-linking after chronic treatment with AngII [16]. Considering these data, it was plausible that LOX transgenesis would result in altered cardiac remodeling after myocardial infarction. However, our present results indicate that this is not the case. In fact, echocardiographic data reveal that myocardial function 28 days after transient coronary occlusion was equally depressed in WT and TgLOX mice. Moreover, both the area of the scar and collagen deposition in distant myocardium were not modified by human LOX transgenesis. This, however, does not mean that LOX does not contribute to scar formation after myocardial infarction. Indeed, we and others have evidenced the up-regulation of LOX in mouse models of myocardial infarct, in both rats and mice [31,32]. Similarly, LOX and LOX-like isoforms have been shown to be transiently increased after injury in a mouse model of permanent ligature of the LAD [33], in which LOX blockade by either the non-selective LOX inhibitor β-aminopropionitrile or a selective LOX neutralizing antibody attenuated collagen accumulation and maturation with a concomitant reduction in ventricular dilatation and an improvement of cardiac function [33]. Our results may simply indicate that the amount of LOX normally present in the cardiac tissue is sufficient enough to assure scar formation after infarction, and that a marked increase in its levels, as occurs in our transgenic model, does not have any additional influence. 

As a limitation of this study, we have not addressed whether LOX transgenesis affects the more severe response triggered by permanent LAD ligation, an approach that led to a stronger fibrotic and hypertrophic response, both processes potentially influenced by LOX [13,16]. However, it should be highlighted that although both permanent and transient LAD occlusion models provide clinically relevant information, only the transient coronary occlusion model allows assessing the impact of blood reperfusion injury, a condition closely resembling the clinical situation of patients subjected to PCI, an intervention that significantly reduces acute mortality after myocardial infarction. 

Isolated cardiofibroblasts from TgLOX mice have been shown to produce higher amounts of collagen I than WT cells, and to express increased levels of α-actin and trasgelin (SM22α), well-recognized markers of the phenotypic transformation of fibroblasts to myofibroblasts [16]. However, they also showed reduced proliferation rates and lower migratory activity [16,34]. Accordingly, it has been reported that myocardial infarction induced by permanent LAD ligation in mice up-regulates fibroblast LOX expression reaching a peak that coincides with the minimal proliferative activity of these cells [35]. Unfortunately, fibroblasts dynamics after transient coronary occlusion has not been assessed, while the relative importance of the impact of LOX on fibroblast proliferation and phenotypic switch under our experimental conditions would be an alternative explanation for our present findings.

## 5. Conclusions

In summary, this study demonstrates that although LOX transgenesis boosts cardiac oxidative stress under basal conditions, LOX over-expression in mice does not result in exacerbated ROS production or altered infarct size and functional recovery in response to acute myocardial ischemia-reperfusion injury, and does not modify post-myocardial infarction remodeling triggered by transient coronary occlusion, a model resembling the clinical scenario of patients with STEMI subjected to PCI.

## Data Availability

The data presented in this study are available within the article and the Supplemental Data.

## Supplementary Materials

The following supporting information can be downloaded at: https://www.mdpi.com/article/10.3390/antiox11010075/s1, Figure S1: Original blots shown in Figure 1B.
