# Supplementary figures and images for: Human Lysyl Oxidase Over-Expression Enhances Baseline Cardiac Oxidative Stress but Does Not Aggravate ROS Generation or Infarct Size Following Myocardial Ischemia-Reperfusion
Source: Antioxidants (Basel). 2021 Dec 29;11(1):75. doi: 10.3390/antiox11010075 (PMC8773108; doi:10.3390/antiox11010075)

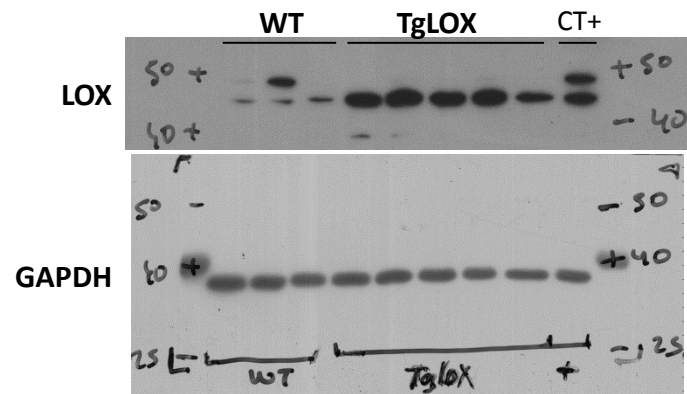

**Figure S1.** Original images corresponding to blots shown in Figure 1B.

Supplement: Supplementary file 1 [file antioxidants-11-00075-s001.zip › antioxidants-1477134-supplementary.pdf]
